# Petunia dihydroflavonol 4-reductase is only a few amino acids away from producing orange pelargonidin-based anthocyanins

**DOI:** 10.3389/fpls.2023.1227219

**Published:** 2023-08-14

**Authors:** Jere Vainio, Saku Mattila, Sara M. Abdou, Nina Sipari, Teemu H. Teeri

**Affiliations:** ^1^Department of Agricultural Sciences, Viikki Plant Science Centre, University of Helsinki, Helsinki, Finland; ^2^Horticulture and Product Physiology Group, Wageningen University, Wageningen, Netherlands; ^3^Viikki Metabolomics Unit, University of Helsinki, Helsinki, Finland

**Keywords:** petunia hybrida, dihydroflavonol 4-reductase, dihydrokaempferol, pelargonidin, GMO, metabolic engineering

## Abstract

Anthocyanins are responsible for the color spectrum of both ornamental and natural flowers. However, not all plant species produce all colors. For example, roses are not blue because they do not naturally possess a hydroxylase that opens the pathway for delphinidin and its derivatives. It is more intriguing why some plants do not carry orange or scarlet red flowers with anthocyanins based on pelargonidin, because the precursor for these anthocyanins should be available if anthocyanins are made at all. The key to this is the substrate specificity of dihydroflavonol 4-reductase (DFR), an enzyme located at the branch point between flavonols and anthocyanins. The most common example is petunia, which does not bear orange flowers unless the enzyme is complemented by biotechnology. We changed a few amino acids in the active site of the enzyme and showed that the mutated petunia DFR started to favor dihydrokaempferol, the precursor to orange pelargonidin, *in vitro*. When transferred to petunia, it produced an orange hue and dramatically more pelargonidin-based anthocyanins in the flowers.

## 1 Introduction

Anthocyanins are best known for their color in flowers and fruits, ranging from orange to red, and purple to blue. Carotenoids and betalains also contribute to the color spectrum in the wild, in flower shops, and at our dinner tables, but anthocyanins dominate. However, these are not the only functions of anthocyanins. These compounds are also stress-induced and help plant tissues cope with abiotic stress (Quina et al., 2009; Silva et al., 2016; Berland et al., 2019). The colors of flowers and fruits are signals to animals that assist plants with their pollination and spread their seeds, activities for which plants offer rewards.

Anthocyanins form a branch of flavonoids, itself a branch of the general phenylpropanoid pathway, which is initiated by the deamination of phenylalanine, or tyrosine in some cases (Stafford, 1991; Wen et al., 2020). Chalcone synthase is the first enzyme dedicated to flavonoid biosynthesis and isomerization of chalcone to naringenin forms the first compound in the flavonoid branch. Although the reaction can also occur spontaneously, it is catalyzed by chalcone isomerase, which exerts stereospecific control over the reaction. Flavonoids are polyphenols containing two aromatic rings. The B-ring is inherited from phenylalanine, and the A-ring is formed from a tetraketide in a reaction catalyzed by chalcone synthase. A heterocyclic C-ring connects the two rings. The B-ring regularly carries a hydroxyl group at position 4′. Additional hydroxylation at position 3′, or 3′ and 5′, forms the three major compound groups of the side branches of the flavonoid pathway in flavones, flavonols, and anthocyanins (**Figure S1**). Further processes of glycosylation, acylation, and methylation result in expansion of each group.

The major anthocyanins can be divided into three groups based on the hydroxylation pattern of the B-ring of their aglycones: pelargonidin with 4′ hydroxylation, cyanidin with 3′,4′ hydroxylation, and delphinidin with 3′,4′,5′ hydroxylation. The aglycones are not stable in the cellular environment but are stabilized through glycosylation and reside in vacuoles (Grotewold and Davies, 2008). These molecules can undergo further transformations, such as acylation and methylation, resulting in thousands of different anthocyanin species across the plant kingdom that are responsible for the entire spectrum of anthocyanin-derived colors in flowers (Koes et al., 2005). However, the structure of anthocyanins is not the only determinant of flower color. Vacuole pH plays an important role, together with copigments (colorless flavonoids that interact with anthocyanins), and metal ions (Aida et al., 2000; Koes et al., 2005).

In ornamental breeding, flower color is one of the main attributes, in addition to agronomical properties such as growth habit, disease resistance, and longevity. Novel colors and color patterns attract customers, and ornamental breeders are constantly searching for novelty. However, there are limitations to the colors of flowers. Interspecific crosses can overcome some of these limitations, which is why many ornamentals, such as petunias and gerberas, are species hybrids with color ranges that exceed those of their original parents. In some cases, limitations can be easily identified as missing parts of the enzymatic arsenal of a species. Blue roses do not exist because roses do not contain the enzyme flavonoid 3′,5′-hydroxylase (F3′5′H) necessary for opening the branch to delphinidin (**Figure S1**). Such limitations can be overcome using biotechnology. By transferring a gene for the missing hydroxylase from another species, “blue” roses, carnations, and chrysanthemums have been bred (Tanaka et al., 1998; Katsumoto et al., 2007; Noda et al., 2017).

The garden petunia, *Petunia hybrida*, is a diploid hybrid of *P. axillaris* and *P. inflata* (Bombarely et al., 2016). Its cultivars have a range of colors, from red to magenta, mauve, and purple, with beautiful petal patterns. However, orange and scarlet red colors are absent and the reason, in this case, is not the missing pattern of B-ring hydroxylation. Orange and scarlet anthocyanins are usually derivatives of the aglycone pelargonidin [or in some cases of cyanidin and delphinidin, like in the wild relative *P. exserta* (Berardi et al., 2021)], which has the simplest B-ring hydroxylation pattern at the 4′ position. Petunia lines homozygous for mutations in both flavonoid 3′-hydroxylase (F3′H) and F3′5′H accumulate only minute amounts of pelargonidin glycosides, whereas the precursor dihydrokaempferol (DHK) accumulates in the plants (Forkmann and Ruhnau, 1987). The reason for the lack of orange color is not a missing enzyme but the substrate specificity of the enzyme dihydroflavonoid 4-reductase (DFR). DHK is a poor substrate for petunia DFR.

Also this obstacle can be overcome by using biotechnology. One of the first examples of metabolic engineering in plants was the transfer of the DFR-encoding gene *A1* from maize to the petunia line RL01, which is a mutant for F3′H and F3′5′H encoding genes. The resulting transgenic line accumulated pelargonin and was described as brick-red in color (Meyer et al., 1987). RL01 is an inbred laboratory line of petunia that is not commercially attractive. Brick red lines were licensed by a breeding company that crossed them with elite garden petunia lines, resulting in lines with bright orange showy flowers (Oud et al., 1995). Oud et al. (1995) reported that orange cultivars with this new trait would enter commercial breeding programs. However, as the EU legislation controlling genetically modified organisms came into effect in 1995, such lines were not commercialized.

Twenty years later, vivid prize-winning orange petunias had appeared in the market (Anonymous, 2016). In the summer of 2015, the city of Helsinki used orange petunias as decorative elements in public places, and flowers with unusual orange scarlet hues attracted our attention. The flowers were not the result of traditional breeding, as they carried the maize DFR-encoding gene, *A1*, along with a kanamycin resistance marker, in an identical configuration to the genetically modified brick red petunias generated in Cologne 30 years earlier (Bashandy and Teeri, 2017; Haselmair-Gosch et al., 2018). It is unclear how these genetically modified lines entered breeding programs and the market; however, they were not authorized in any country and had to be removed. As breeders legally use commercial cultivars as crossing parents, not all unauthorized cultivars doomed for destruction were orange in color (Anonymous, 2022).

Although the orange-scarlet petunias turned out to be transgenic lines, it might still be possible for breeders to find a natural mutant of the DFR-encoding gene that would allow the enzyme to reduce DHK and initiate pelargonidin biosynthesis. It is argued that a single nucleotide change would not be sufficient, as thousands of petunia breeders who have observed millions of flowers would have found such a mutation (Johnson et al., 2001). The mutation frequency per base pair and generation is 7×10^-9^ in arabidopsis (Ossowski et al., 2010). Therefore, the assumed frequency of finding a mutation in a particular base of the petunia DFR is in the range of one in 100 million plants. This mutation, however, would need to be in a particular genetic background to be identified.

By concentrating on a 26 amino acid long stretch, which is believed to be the key to DFR substrate specificity (Johnson et al., 2001), and making simple educated guesses, we show that a change in only one or two amino acid residues is sufficient to allow efficient DHK reduction by petunia DFR.

## 2 Materials and methods

### 2.1 Mutagenesis and vector construction

The isolation of petunia cDNA for *DFRA* from a blue-flowered cultivar and the generation of the Gateway entry vector were described by Zhu et al. (2023). The sequence has been deposited in GenBank under the accession number MW929212. Mutagenesis was performed in the entry vector using primers carrying the intended changes (**Supplemental Table** **S4**). First, thirty cycles of linear PCR (1 min 94°C, 30 s 55°C, 8 min 72°C) was performed separately with each of the two primers using 500 ng plasmid, 0.4 µM primer, 0.2 mM dNTPs (each) and Phusion high fidelity DNA-polymerase (Thermo-Fischer) in 25 µL. Equal volumes of the amplified single stranded molecules were mixed, heated 5 min at 98°C and allowed to cool to 50°C in ca. ten minutes. After digesting the sample with DpnI to remove unmutated plasmid, it was transformed to *E. coli* DH5α.

After verification by sequencing, the inserts were transferred from the entry vectors to the destination vectors in an LR reaction according to the Gateway manual (Invitrogen), to pK2GW7 (Karimi et al., 2002) for stable transformation by agrobacterium, and to pEAQ-HT-DEST1 (Peyret and Lomonossoff, 2013) for agroinfiltration. The resulting expression vectors were verified using restriction enzymes and transformed into *Agrobacterium tumefaciens* C58C1(pGV2260) (Deblaere et al., 1985) using the freeze-thaw method (Wise et al., 2006).

### 2.2 Transient expression in *Nicotiana benthamiana* and assay for DFR activity

Transient expression of DFR was performed in the top leaves of 6-week-old *N. benthamiana* plants according to Bashandy et al. (2015) and Zhu et al. (2023). Expression was allowed to continue for three days, after which 100 mg of leaf tissue was harvested in liquid nitrogen and milled to powder with an Oscillating Mill MM400 (Retsch, Germany). Extraction buffer (450 µL; 100 mM Tris-Cl pH 7.5, 0.2% 2-mercaptoethanol and a cocktail of protease inhibitors (Complete Mini EDTA free, Roche)) was added to frozen tissue powder, the thawed homogenate was kept on ice for 15 min and cleared twice in a microcentrifuge (full speed, 4°C).

DFR assay was performed as described by Zhu et al. (2023). Briefly, 10 µL of *N. benthamiana* extract was used in an assay of volume 100 µL, comprising a final concentration of 79 mM MOPS-KOH, pH 7.0, 1 mM NADPH, 1% DMSO, and 100 µM DHK, dihydroquercetin (DHQ), or dihydromyricetin (DHM). After 10 min at 30°C, the reaction was terminated by adding 20 µL glacial acetic acid. The sample was then extracted with 163 µL and 150 µL ethyl acetate, combined and dried. The dried extracts were dissolved in BuOH-HCl (95 parts *n*-butanol and 5 parts 37% HCl, supplemented with 1/30 volume of 2% NH_4_Fe(SO_4_)_2_ ·12H_2_O in 2 N HCl) and heated for 30 min at 95°C. The absorbance of the butanoylated product was measured at 535, 550, and 560 nm for DHK, DHQ, and DHM, respectively, corrected by subtracting the absorbance at 660 nm and converted to µM substrate used by multiplying with 62.9, 76.6, and 91.7 for DHK, DHQ, and DHM, respectively (Zhu et al., 2023).

(2R,3R)-dihydrokaempferol (DHK), (2R,3R)-dihydroquercetin (DHQ) and (2R,3R)-dihydromyricetin (DHM) and pelargonidin were purchased form TransMIT PlantMetaChem (Giessen, Germany) and other chemicals from Sigma-Aldrich unless specified otherwise.

**Figure 1:**
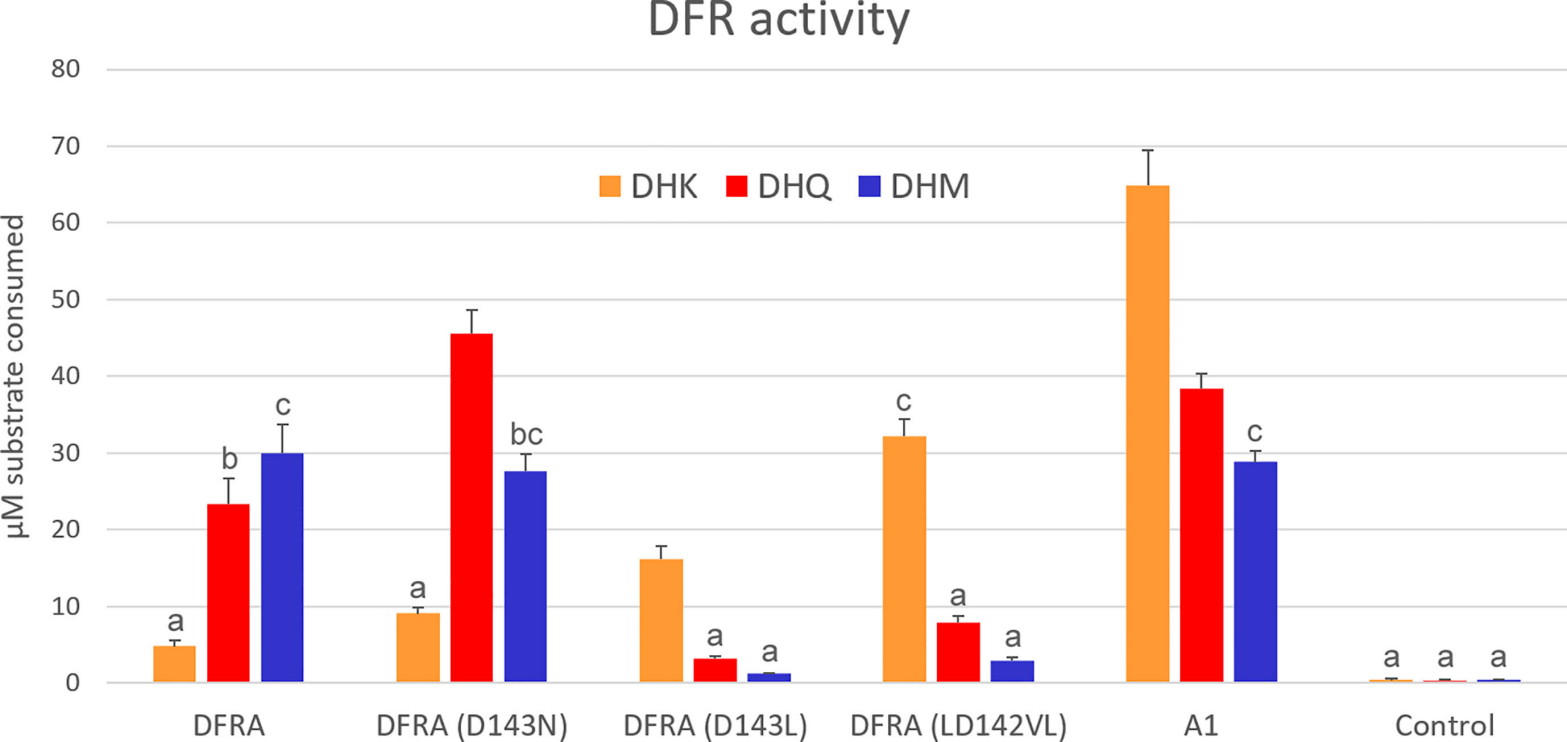
*In vitro* enzymatic activity of DFR expressed in tobacco cells. Crude protein extracts were assayed with 100 µM dihydrokaempferol (DHK), dihydroquercetin (DHQ), or dihydromyricetin (DHM), and 1 mM NADPH for 10 min. DFRA is the petunia enzyme and A1 is DFR from maize. The control is extracted from an uninfiltrated plant. Error bars show standard deviations of the four biological replicates. Pairs of values that do not share an alphabet are significantly different according to Tukey’s HSD Test (p<0.05).

For the assays shown in **Figure 1**, two leaves of two plants from each construct were infiltrated and collected three days after infiltration. Samples from the two plants were assayed on separate days, and for each extract, the assay was repeated at 30 min intervals between the assays. The technical repeats were averaged and the four biological replicates were statistically evaluated using ANOVA with *post-hoc* Tukey HSD Test.

### 2.3 Petunia transformation

The petunia line M61×W80 (Strazzer et al., 2023) is a mutant for the F3′H and F3′5′H encoding genes (*ht1 ht1*, *hf1 hf1*, and *hf2 hf2*) but heterozygous for a functional DFR-encoding gene (*DFRA dfra*). Transformation of M61×W80 was performed as described by Conner et al. (2009). In brief, greenhouse-grown petunia leaves were surface sterilized for 1 min in 70% ethanol, washed with sterile water, and incubated for 10 min in one-quarter-strength household bleach. After washing five times with sterile water, the leaves were cut into 5 × 5 mm^2^ squares and infected for 5 min with an Agrobacterium suspension (1/7 diluted overnight culture). After blotting with sterile filter paper, the leaf pieces were placed on MS medium with vitamins (Duchefa Biochemie, Netherlands) supplemented with 20 g/L sucrose, 10 g/L glucose, 1 mg/L folic acid, 1 mg/L 6-benzylaminopurine, and 0.1 mg/L 1-naphthaleneacetic acid, and solidified with 0.8% agar. After 3 d at 23°C in the dark, the leaf pieces were transferred to a selection medium containing, in addition, 100 mg/L kanamycin and 100 mg/L ticarcillin and clavulanic acid mixture (Duchefa Biochemie). Leaf disks were grown at 23°C under fluorescent light and transferred to fresh plates every two weeks until shoots emerged, which were rooted in the same medium but half strength, without hormones and kanamycin, and subsequently transferred to the growth room with fluorescent lights, temperature of 23°C, and daylength of 16 hours. Plants were grown in peat:vermiculite mixture (1:1) and fertilized with commercial fertilizer (Substral, Germany).

Five to fifteen independent transgenic lines were recovered for each mutant construct and analyzed for anthocyanidin content (**Table S1**). The first flowers were discarded before the analysis started.

### 2.4 Anthocyanin extraction and liquid chromatography

For anthocyanidin analysis, petal limbs of each flower were cut, weighed, and crushed in liquid nitrogen. Anthocyanins were extracted using five volumes of 80% methanol and 1% concentrated HCl by sonication for 15 min. After clearing by centrifugation, the extract was mixed 1:1 with 4 N HCl and heated for one hour at 95°C to hydrolyze them to anthocyanidins (the aglycones).

HPLC analysis was performed using an Agilent 1100 Series HPLC system (Agilent, CA, USA) equipped with a Zorbax Rc-C18 column (Agilent). The mobile phases were 0.08% trifluoroacetic acid (TFA) in water and 0.08% TFA in acetonitrile and a linear gradient from 10–90% acetonitrile was run for 20 min at a flow rate of 1 mL/min. The analytes were monitored at 525 nm, and the peak areas were reported using the Agilent software. For estimating the amount of pelargonidin in the samples, purified pelargonidin was dissolved in methanol with 1% concentrated HCl and diluted to 2.5-20 µg/ml for a standard curve (**Table S1**).

High-resolution mass spectrometry (UPLC-PDA-HDMS) analyses of the petunia samples were performed in the Viikki Metabolomics Unit at the University of Helsinki, Finland. Waters Synapt G2 HDMS mass spectrometer (Waters, Milford MA, USA) was used with the Waters Acquity UPLC system (Waters) via an ESI source with an additional diode array detector (Acquity PDA, Waters). The mass range was set from 90–800 with a 0.1 s scan rate in HDMS, while a wavelength range of 210–499 nm with a scan rate of 40 points/s was used in the PDA detector. Samples were analyzed in positive, sensitivity ion mode (ESI+), with a capillary voltage of 3.0 kV, source temperature of 120°C, desolvation temperature of 360°C, cone gas flow rate of 20 L/h, and desolvation gas flow rate of 800 L/h.

The secondary metabolites were separated on an Acquity BEH C18 UPLC column (1.7 µm, 50 × 2.1 mm, Waters, Ireland) at 40°C. The mobile phase consisted of water (A) and acetonitrile (B) (Chromasolv grade) both containing 0.1% formic acid. A linear gradient of eluents was used as follows: the gradient started at 95% A and was decreased to 10% over 10 min. Then, the ratio was switched back to 95% at 10.1 min and left to equilibrate for 1 min, with a total run time of 11 min. The injection volume was 3 µL and the flow rate of the mobile phase was 0.6 mL/min. The temperature of the sample tray was set at 10°C.

### 2.5 Petunia DFR 3D structure prediction and modeling of substrate interactions

Computational modeling of the structures of wild-type DFRA and its mutants was performed using ColabFold (Mirdita et al., 2022), an online version of AlphaFold2 (Jumper et al., 2021). Relaxed versions of the top-ranked models were used in interaction studies. Additionally, the double mutant LD142VL was prepared by manual amino acid replacement using UCSF ChimeraX (Pettersen et al., 2021), with no significant binding pocket structure changes. Ligand-binding modeling tools, such as DiffDock (Corso et al., 2022) have also been utilized to map ligand-binding sites. Unfortunately, DiffDock could not identify the exact binding sites for primary substrates such as DHM. A comparison of the binding sites of the solved *Vitis vinifera* structure revealed that the NADPH-binding site was better established than that of the primary substrates. Dihydroflavonol substrates were positioned manually, with substrate-enzyme interactions with nearby residues mimicking those in the existing crystal structure (Petit et al., 2007). Manual ligand-binding interaction prediction and molecular graphic analysis were performed using ChimeraX (Pettersen et al., 2021).

## 3 Results

### 3.1 Design of mutants

To examine the possibility of altering the substrate preference of the petunia DFR with a minimum number of amino acid changes, we analyzed previous results addressing this. In a classical study addressing the substrate specificity of the petunia DFR, encoded by *DFRA*, Johnson et al. (2001) constructed hybrids between *DFRA* and a gerbera gene encoding DFR. The substrate specificity of the hybrid enzymes was assessed by transferring the hybrid genes to RL01, the same petunia laboratory line used by Meyer et al. (1987). The gerbera gene is a cDNA molecule representing the allele *GDFR1-1* that encodes an enzyme with a strong preference for DHK (Zhu et al., 2023). Petunia DFR has a preference for DHM (three hydroxyl groups, **Figure S1**), and accepts DHQ but poorly accepts DHK (Haselmair-Gosch et al., 2018).

Johnson et al. (2001) identified a stretch of 26 amino acids that switched the petunia-type substrate preference to the gerbera type, leading to pelargonin production in transgenic RL01. Compared with five enzymes that allow pelargonidin biosynthesis (DFR from gerbera, rose, snapdragon, carnation, and maize), the petunia DFR is unique at four positions. One is aspartate (D) at position 143 (petunia numbering), where the other enzymes have asparagine (N). This is considered by many the key amino acid for substrate specificity (Johnson et al., 2001; Chu et al., 2014; Berardi et al., 2021).

Johnson et al. (2001) made several mutations in the gerbera DFR by introducing hydrophobic amino acids in place of hydrophilic ones in the 26-amino-acid long stretch and identified a change (N134L, gerbera numbering, corresponding to position 143 in petunia numbering) that further increased the gerbera enzyme preference for DHK based on their *in vivo* assay.

Based on this information, we made three mutations in the petunia *DFRA* gene (**Figure S2**). First, we mutated the putative substrate determining aspartic acid residue to asparagine, D143N (petunia numbering). Second, to mimic the enhanced pattern of DHK use in GDFR1-1 identified by Johnson et al. (2001), we introduced leucine at the same position, D143L. Third, to bring the petunia DFR a step closer to GDFR1-1, which prefers DHK, we mutated additionally the prior amino acid from leucine to valine, LD142VL.

### 3.2 Expression of DFR in *N. benthamiana* and *in vitro* assays of enzymatic activity

In order to assay the DFR enzymes for their substrate preference, the wild-type and mutated petunia *DFRA* sequences, along with the maize *A1* cDNA encoding DFR, were expressed in *N. benthamiana* using agroinfiltration (Zhu et al., 2023).

Tobacco leaves were extracted three days after infiltration and assayed for DFR activity using the BuOH-HCl method originally described by Porter et al. (1986) and refined by Zhu et al. (2023). Wild-type petunia DFR prefers DHM and exhibits low activity with DHK. While the D134N mutation draws substrate preference towards less-hydroxylated dihydroflavonols, it does not induce a dramatic change in DHK acceptance. In contrast, the D134L mutation inverts the substrate preference to favor DHK and rejects DHM. The LD142VL mutation further improved DHK use, making the enzyme DHK specific. Maize DFR A1 showed a preference for DHK, but accepted well also DHQ and DHM (**Figure 1**).

### 3.3 Expression of DFR in transgenic petunia and formation of pelargonidin

For stable expression in transgenic petunia and to observe possibly increased pelargonidin production *in planta*, the DFR-encoding genes were expressed from a vector where the transgene is driven by a 35S promoter. The host for the transgenes was the petunia line M61×W80, which is a cross between two inbred petunia laboratory lines (Strazzer et al., 2023). The line harbored mutations in genes encoding F3′H and F3′5′H but a wild-type *DFRA* allele. The transgenic lines (and the non-transgenic control) were analyzed for the pattern of anthocyanidins in their corolla limbs by extracting anthocyanins, hydrolyzing them into their aglycones, and separating the aglycones using HPLC.

**Figure 2:**
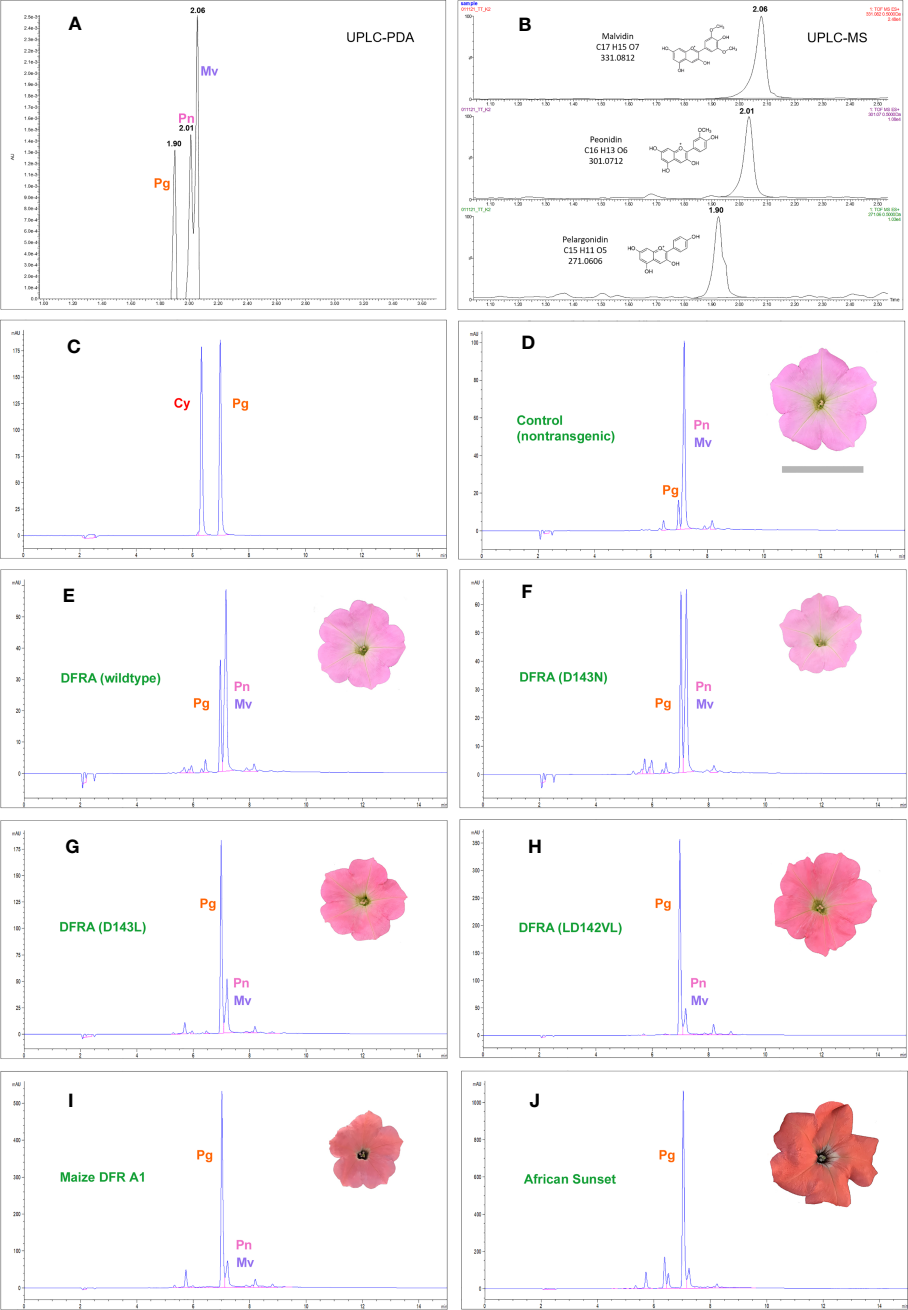
Chromatograms of anthocyanidins extracted from petunia petal limbs. The anthocyanidins in the petunia line M61×W80, used for transformation in this work, resolve into three peaks in UPLC **(A, B)** that were identified as pelargonidin (Pg), peonidin (Pn), and malvidin (Mv) by mass spectrometry. In HPLC **(D–I)**, peonidin and malvidin do not resolve chromatographically and are counted as one reference peak in the samples. M61×W80 has a minute amount of pelargonidin-derived anthocyanins **(D)** that is increased in all transgenic lines expressing the petunia *DFRA* gene and its mutants under the 35S promoter. Examples are from transgenic lines expressing the *DFRA* wild-type **(E)** and the D143N **(F)**, D143L **(G)**, and LD142VL **(H)** mutants. **(I)** shows the M61×W80 line transformed with the maize gene *A1* encoding DFR and **(J)** shows the *A1* carrying commercial variety African Sunset. Cyanidin (Cy) and pelargonidin are shown in **(C)** for comparison.

The flowers of non-transgenic M61×W80 appeared pink because of the accumulation of a small amount of anthocyanins derived from peonidin and malvidin, and a minute amount from pelargonidin (**Figures 2A–C**). To compensate for the variability in the anthocyanin content of flowers produced and analyzed at different times, the ratio of the peak areas of pelargonidin to those of peonidin-malvidin (which was not chromatographically resolved in our HPLC) was calculated (rPg). Many transgenic lines did not express the transgene (or escaped kanamycin selection during transformation), and lines with rPg less than the highest value observed in the nontransgenic M61×W80 control (0.41) cannot be distinguished from non-expressors or escapes (see **Supplemental Tables S1–S3**).

Transgenic lines expressing wild-type *DFRA* increased the pelargonidin content of the petals by a small amount, which is in line with the fact that the enzyme accepts DHK at a low rate. All the mutant DFRA enzymes led to elevated pelargonidin levels. The best mutant was LD142VL; however, it still fell short of the maize A1 DFR in producing pelargonidin (**Figure 2**; **Tables S1–S3**). Another approach to evaluate transgenic lines is to score the highest pelargonidin producer for each *DFRA* mutant, similar to how a breeder selects the best-performing line for production or further crossing. For the D143N mutant, the amount was modest, with 18 µg/g fresh weight of the petal limb, but the LD142VL mutant led to 146 µg/g, which was more than half of the best transformant carrying the maize *A1* gene at 251 µg/g. The transgenic petunia cultivar African Sunset, carrying a 35S-*A1* construct and bred for its intense orange color, is shown as a control and accumulates pelargonidin up to 329 µg/g fresh weight (**Figure 2**; **Tables S2, S3**).

### 3.4 Mutations in the DFR 3D structure

**Figure 3:**
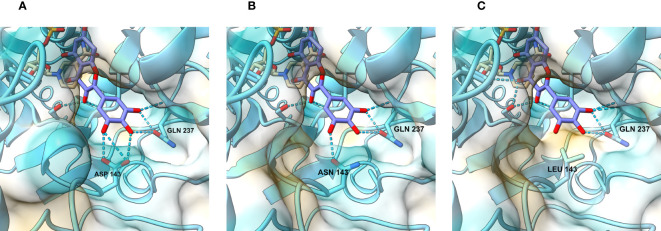
View of the catalytic site and the regions responsible for substrate recognition. In the wild-type petunia DFRA **(A)**, hydrogen bond contacts are established between the DHM B-ring hydroxyl residues, aspartate (D143), and glutamine (Q237). When D143 is replaced by asparagine **(B)** in the D143N mutant, interactions with the B-ring hydroxyl groups remain. In the mutants where the aspartate residue is replaced with an aliphatic residue **(C)**, such as leucine, these interactions disappear and are replaced by hydrophobic repulsion.

With the intention of gaining insight into how the mutations we made change the interaction between the enzyme and its substrates, we modelled the active site of the wild type and mutated petunia DFR. The only DFR this far crystallized for 3D structure determination is the grapevine (*Vitis vinifera*) VvDFR, which accepts DHQ as a substrate (Petit et al., 2007). We used ColabFold (Mirdita et al., 2022), a web-based version of Alphafold2 (Jumper et al., 2021), to predict the 3D structure of the petunia enzyme. As shown in **Figure 3A**, the DHM in the model is positioned analogous to the DHQ in the grapevine crystal structure (Petit et al., 2007). In these computational models, the interactions between the B-ring hydroxyls of the substrate and the enzyme occur between aspartate (D143) and glutamine (Q237). Both D at position 143 (**Figure 3A**) and N (**Figure 3B**) at the corresponding position in VvDFR can interact with the substrate B-ring hydroxyls through hydrogen bonding, giving no obvious clue as to why the mutation D143N in *DFRA* would increase activity with DHK and DHQ. However, replacing the polar amino acid with a nonpolar one in D143L (**Figure 3C**) abolished this interaction, considerably dropping the enzyme activity with DHQ and DHM, whereas the activity with the less polar DHK increased (hypothetically due to an increased hydrophobic environment and 3′,5′-hydroxyl repulsion). Further replacement of the L at position 142 with V (mutation LD142VL) makes nearly no change in the active site and the modelling does not help in explaining the increased activity with all substrates.

## 4 Discussion

### 4.1 DFRA is a few amino acids away from accepting DHK

We created mutations that changed the amino acids in a critical position of the petunia dihydroflavonol 4-reductase that interact with the B-ring of the dihydroflavonol substrate according to the 3D structure of the grapevine DFR. Based on sequence comparisons, it has been suggested that the aspartic acid at position 143 of the petunia enzyme is an important determinant of the poor use of DHK. However, replacing aspartic acid with asparagine, which is found at the corresponding position in enzymes that accept DHK, had little effect. However, a hydrophobic leucine at this position almost completely abolished the use of DHQ and DHM while improving the use of DHK. By additionally changing the preceding leucine to valine, the petunia DFR was converted from DHK avoider to DHK favorer.

We further showed that when expressed from a 35S promoter in transgenic petunia, the mutant enzymes led to pelargonidin biosynthesis in the M61×W80 background, which accumulates DHK because of mutations in the hydroxylases responsible for DHQ and DHM biosynthesis (F3′H and F3′5′H, respectively). The intensity of the orange color of our transgenic lines was not comparable to that of the commercial cultivars that carried the maize DFR-encoding gene *A1*; however, they did compare well with the M61×W80 control transformants with 35S-*A1* (**Figure 2**).

### 4.2 Breeding for orange petunias

Similar to the lines produced with *A1* in Cologne (Meyer et al., 1987; Oud et al., 1995), crossing the laboratory lines carrying the *DFRA* mutants with commercial varieties could result in a significant improvement in color intensity. Even better, targeted mutagenesis using gene editing of the DFR-encoding gene residing in the petunia genome may yield stable expression of the altered phenotype in one step. The D143N mutation is a single-base mutation that is not considered a genetic modification in many countries. However, as we demonstrated, the change in substrate preference is subtle. To obtain the best results, several nucleotides must be modified. D143L was achieved with two nucleotide changes and LD142VL with three changes. Techniques for this type of targeted mutagenesis include oligonucleotide mutagenesis in protoplasts (Beetham et al., 1999) and CRISPR/Cas-based prime editing (Anzalone et al., 2019). According to the current legislation, these multiple changes would cause the plants to be classified as genetically modified organisms. In the EU, even single nucleotide changes with gene editing techniques are considered genetically modified and are strictly regulated. Very recently, the European Commission’s legal proposal for new genomic techniques includes that simple edits, including up to 20 nucleotide substitutions, would be considered to be equivalent to conventional plants (Anonymous, 2023). Also, the rapid deregulation of the originally illegal orange petunia in the US (Anonymous, 2021) could indicate a more positive turn in public attitudes toward new tools in the breeder’s toolbox, at least for breeding non-food crop plants.

### 4.3 Orange is attractive–but does not give stress protection

In addition to human consumers (Anonymous, 2016), orange and scarlet red flowers attract bird pollinators (Rodríguez-Gironés and Santamaría, 2004). However, pelargonidin-based anthocyanins, which are typical of orange and scarlet red, do not provide the protective functions of these compounds. Plants that harbor pelargonins in their flowers still synthesize (stress-induced) cyanidin derivatives in their vegetative parts. Examples can be found in gerbera (Deng et al., 2014) or in the pelargonium itself (unpublished observations). Assays of antioxidant capacity have shown that pelargonidin is a weaker antioxidant than cyanidin or delphinidin, and vicinal hydroxyl groups seem to be important for this function (Rice-Evans et al., 1996; Wang et al., 1997). Strong activity of the hydroxylases F3′H and F3′5′H does not allow substantial formation of pelargonidin, but there are plants, like petunia, that have evolved to additionally control this through the substrate preference of the DFR enzyme. Besides DFRA, enzymes that avoid reduction of DHK have been identified in many species, including Cymbidium orchids, strawberries, and arabidopsis (Johnson et al., 1999; Leonard et al., 2008; Miosic et al., 2014).

## Data availability statement

The datasets presented in this study can be found in the article/**Supplementary Material**.

## Author contributions

JV, SM and TT designed the research, JV, SM, SA, NS and TT carried out the experiments, JV and SM did the computer modelling, JV, SM and TT analyzed the results, TT, JV and SM wrote the manuscript with input from all authors. All authors read and approved the final manuscript.
