# Supplementary material for: Petunia dihydroflavonol 4-reductase is only a few amino acids away from producing orange pelargonidin-based anthocyanins
Source: Front Plant Sci. 2023 Aug 14;14:1227219. doi: 10.3389/fpls.2023.1227219 (PMC10461392; doi:10.3389/fpls.2023.1227219)
Supplement: Supplementary file 1 [file DataSheet_1.pdf]

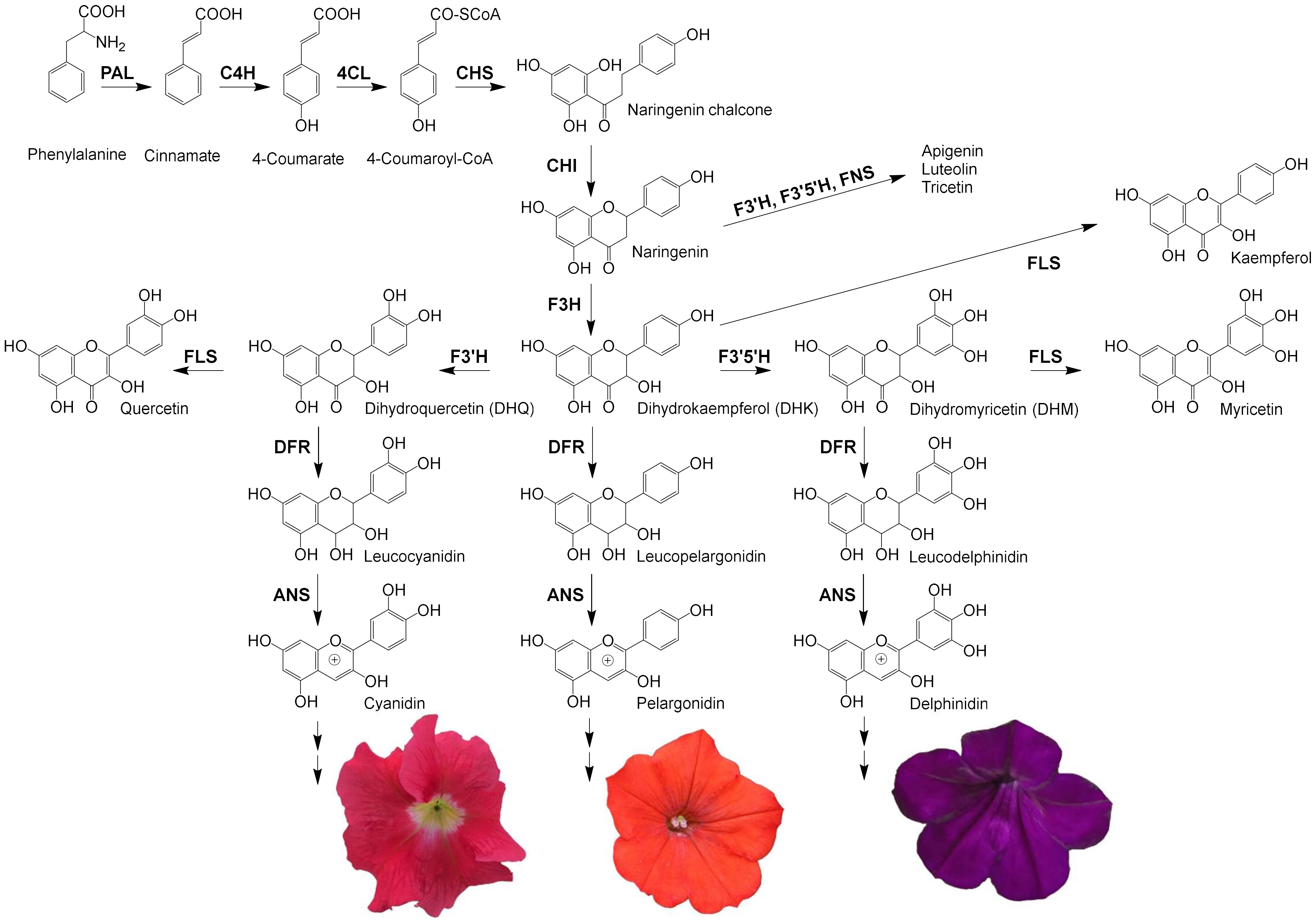

Figure 1. Pathway to the colored anthocyanidins. Petunia flowers containing orange pelargonidin derivatives do not occur naturally due to the substrate specificity of petunia DFR. **PAL**, phenylalanine ammonialyase; **C4H**, cinnamate-4-hydroxylase; **4CL**, 4-coumaryl-CoA ligase; **CHS**, chalcone synthase; **CHI**, chalcone-flavanone isomerase; **F3H**, flavanone 3-hydroxylase; **F3'H**, flavonoid 3'-hydroxylase; **F3'5'H**, flavonoid 3',5'-hydroxylase; **DFR**, dihydroflavonol 4-reductase; **ANS**, anthocyanidin synthase.

```
CLUSTAL O(1.2.4) multiple sequence alignment

GDFR1-1      ---------MEEDSPATVCVTGAAGFIGSWLVMRLLERGYVVHATVRDPGDLKKVKHLLE    51
                          :  ************************ ******** : ********
DFRA         MASEAVHAPSPPVAVPTVCVTGAAGFIGSWLVMRLLERGYNVHATVRDPENKKKVKHLLE    60

GDFR1-1      LPKAQTNLKLWKADLTQEGSFDEAIQGCHGVFHLATPMDFESKDPENEIIKPTIEGVLSI    111
             ****:***.******* ***********:****:**************:****:.*:***
DFRA         LPKADTNLTLWKADLTVEGSFDEAIQGCQGVFHVATPMDFESKDPENEVIKPTVRGMLSI    120

                                 134
GDFR1-1      IRSCVKAKTVKKLVFTSSAGTVNGQEKQLHVYDESHWSDLDFIYSKKMTAWMYFVSKTLA    171
             *.**.**:***:*********:: **:*  .**:: ********:****.****.** **
DFRA         IESCAKANTVKRLVFTSSAGTLDVQEQQKLFYDQTSWSDLDFIYAKKMTGWMYFASKILA    180
                                 143
                                   D143N
                                   D143L    Mutations in DFRA
                                  LD142VL

GDFR1-1      EKAAWDATKGNNISFISIIPTLVVGPFITSTFPPSLVTALSLITGNEAHYSIIKQGQYVH    231
             **** : :* :**.****** ******** ******:*************.*********
DFRA         EKAAMEEAKKKNIDFISIIPPLVVGPFITPTFPPSLITALSLITGNEAHYCIIKQGQYVH    240

GDFR1-1      LDDLCECHIYLYENPKAKGRYICSSHDATIHQLAKIIKDKWPEYYIPTKFPGIDEELPIV    291
             ******.**:***:***.**:*****.* *:::**::::******:**:* ***::**:*
DFRA         LDDLCEAHIFLYEHPKADGRFICSSHHAIIYDVAKMVREKWPEYYVPTEFKGIDKDLPVV    300

GDFR1-1      SFSSKKLIDTGFEFKYNLEDMFKGAIDTCREKGLLPYSTIKNHINGNHVNGVHHYIKNND    351
             ******* * **:***.****:********:* ***:** . . **:: :.:    :*
DFRA         SFSSKKLTDMGFQFKYTLEDMYKGAIDTCRQKQLLPFSTRSAEDNGHNREAIAISAQNYA    360

GDFR1-1      DDHEKGLLCCSKEGQ-----     366
             ..:*:. :.  .*
DFRA         SGKENAPVANHTEMLSNVEV     380
```

Figure S2. Alignment of the gerbera **GDFR1-1** and petunia **DFRA** amino acid sequences with Clustal Omega sequence alignment program (https://www.ebi.ac.uk/Tools/msa/clustalo/). GDFR1-1 prefers dihydrokaempferol (DHK) as substrate and reduces it to leucopelargonidin while DFRA has very low activity with this substrate. The 26 amino acids long stretch identified by Johnson et al. (2001) to be important for the difference in substrate specificity is boxed and marked in blue font. Below the box, mutations described in this paper are marked, aligned with the aspartate residue (**D**) at position 143 in DFRA. The corresponding position in gerbera is 134.
